# Compartmentalization of Total and Virus-Specific Tissue-Resident Memory CD8+ T Cells in Human Lymphoid Organs

**DOI:** 10.1371/journal.ppat.1005799

**Published:** 2016-08-19

**Authors:** Heng Giap Woon, Asolina Braun, Jane Li, Corey Smith, Jarem Edwards, Frederic Sierro, Carl G. Feng, Rajiv Khanna, Michael Elliot, Andrew Bell, Andrew D. Hislop, Stuart G. Tangye, Alan B. Rickinson, Thomas Gebhardt, Warwick J. Britton, Umaimainthan Palendira

**Affiliations:** 1 Centenary Institute, The University of Sydney, Newtown, New South Wales, Australia; 2 Department of Microbiology and Immunology, The University of Melbourne, at the Peter Doherty Institute for Infection and Immunity, Melbourne, Victoria, Australia; 3 QIMR Berghofer Medical Research Institute, Brisbane, Queensland, Australia; 4 Discipline of Infectious Diseases and Immunology, Sydney Medical School, The University of Sydney, Newtown, New South Wales, Australia; 5 Chris O’Brien Lifehouse Cancer Centre, Royal Prince Alfred Hospital, Camperdown, Sydney, New South Wales, Australia; 6 Sydney Medical School, The University of Sydney, Newtown, New South Wales, Australia; 7 School of Cancer Sciences and MRC Centre for Immune Regulation, University of Birmingham, Edgbaston, United Kingdom; 8 Garvan Institute of Medical Research, Darlinghurst, New South Wales, Australia; 9 St Vincent’s Clinical School, University of New South Wales, Darlinghurst, New South Wales, Australia; University of Zurich, SWITZERLAND

## Abstract

Disruption of T cell memory during severe immune suppression results in reactivation of chronic viral infections, such as Epstein Barr virus (EBV) and Cytomegalovirus (CMV). How different subsets of memory T cells contribute to the protective immunity against these viruses remains poorly defined. In this study we examined the compartmentalization of virus-specific, tissue resident memory CD8^+^ T cells in human lymphoid organs. This revealed two distinct populations of memory CD8^+^ T cells, that were CD69^+^CD103^+^ and CD69^+^CD103^—^, and were retained within the spleen and tonsils in the absence of recent T cell stimulation. These two types of memory cells were distinct not only in their phenotype and transcriptional profile, but also in their anatomical localization within tonsils and spleen. The EBV-specific, but not CMV-specific, CD8^+^ memory T cells preferentially accumulated in the tonsils and acquired a phenotype that ensured their retention at the epithelial sites where EBV replicates. *In vitro* studies revealed that the cytokine IL-15 can potentiate the retention of circulating effector memory CD8^+^ T cells by down-regulating the expression of sphingosine-1-phosphate receptor, required for T cell exit from tissues, and its transcriptional activator, Kruppel-like factor 2 (KLF2). Within the tonsils the expression of IL-15 was detected in regions where CD8^+^ T cells localized, further supporting a role for this cytokine in T cell retention. Together this study provides evidence for the compartmentalization of distinct types of resident memory T cells that could contribute to the long-term protection against persisting viral infections.

## Author Summary

Some viruses have the capacity to establish chronic infections in humans. How different T cell populations effectively control these infections has not been clear. Continuous circulation of memory T cells was thought to be crucial for effective immune surveillance against such infections. Recent studies in mice however, have shown that non-circulating tissue resident memory populations can also contribute to protective immunity. In this study we have examined the distribution, localization and characteristics of Epstein-Barr virus and Cytomegalovirus-specific T cells in different human tissues. This showed that virus-specific T cells were differentially distributed in different tissues and there was preferential accumulation of EBV-specific resident memory T cells at sites where EBV reactivates. *In vitro* studies showed that IL-15 and TGF-β could cooperate to extinguish tissue exit signals in T cells and therefore potentiate their retention within tissues. IL-15 expression was also detected in areas where T cells aggregated within the tissue. Together our study provides insight into how distinct memory T cells are compartmentalized in tissues to maintain long-term protection against persisting viral infections.

## Introduction

It has recently become evident that protective T cell immunity relies not only on circulating memory T cells but also on non-circulating resident memory populations [1–5]. These resident memory T (Trm) cells have been identified in a variety of different non-lymphoid and lymphoid tissues in mice [6–10]. Importantly, when compared to their circulating counterparts Trm cells provide superior protection against reinfection at their site of localisation [6,11–16]. The characteristics of these cells in humans however, are poorly understood. A greater understanding of the mechanisms that regulate their development and maintenance is paramount for future vaccine strategies.

Residence within tissue environments depends upon the ability of T cells to overcome the egress signals. This is achieved by acquiring expression of receptors that enhance cellular interaction within the tissue and facilitate survival for prolonged periods within that tissue. The exit signal for T cells is largely mediated by the concentration gradient of sphingosine-1-phosphate and expression of its receptor, S1P1, on T cells [17]. Accordingly, studies in mice have shown that Trm cells completely lack the expression of S1P1 as well as its transcriptional regulator KLF2 [18,19]. T cell exit through the efferent lymphatic system is facilitated by the expression of CCR7. KLF2 is also known to positively regulate the transcription of CCR7 [20], and therefore the loss of KLF2 may also abrogate the CCR7 mediated T cell exit. Trm cells are further distinguished from their circulating counterparts by constitutively expressing CD69. This C-type lectin has traditionally been considered as a marker of T cell activation, but its role in promoting tissue residence, through binding to and down-modulating pre-existing S1P1 on the T cell surface, has only recently been recognized [21–23]. A second surface marker associated with tissue residence is CD103, the alpha chain of the integrin αEβ7 which mediates T cell binding to E-cadherin expressed on epithelial tissues [24]. In the mouse, at least two distinct subsets of Trm cells have been identified based on the presence or absence of CD103, with the CD103^+^ subset largely found at barrier surfaces [6,13,25–27].

To date, studies in man suggest that Trm cells are likely to be present in both lymphoid and non-lymphoid organs [28–32]. A recent study has shown that human skin is populated with at least two distinct memory T cell subsets that are non-circulating resident populations. This was demonstrated in patients who underwent alemtuzumab treatment, which selectively depleted the circulating T cell populations [29]. Most other studies however, have relied solely on the expression of CD69 as a Trm marker and, while large numbers of CD69^+^CD8^+^ T cells have been reported in human lymphoid organs [28,30], the significance of such findings is difficult to judge. Firstly, it is not clear whether these CD69^+^CD8^+^ T cells possess other aspects of the Trm phenotype, such as loss of S1P1 and KLF2, or whether they merely express CD69 as a result of recent activation. Furthermore a crucial feature attributed to Trm cells in mouse models, namely the ability of antigen-specific cells to persist at sites of potential antigen encounter [10], has only been examined in human skin [33] or lungs [34]. Whether antigen-specific Trm cells accumulate in human lymphoid tissues is unknown.

In that context, it is relevant to compare the distribution of CD8^+^ T cells to two common human herpesviruses, Epstein-Barr virus (EBV) and cytomegalovirus (CMV) in different human tissues. Both elicit numerically strong CD8^+^ T cell responses, but are harboured at different sites *in vivo* [35,36]. EBV persists as a latent infection of a memory B cell population that preferentially recirculates between the blood and oropharyngeal lymphoid tissues such as the tonsil [37]. Occasional reactivation from the latent reservoir is thought to seed foci of lytic infection in oropharyngeal epithelium, leading to periods of viral shedding into throat washings [36]. By contrast, CMV persists as a latent infection of the myeloid lineage with the capacity to reactivate to lytic infection at various tissue sites [35]. In this study we have focused on two human lymphoid tissues, namely spleen and tonsils, and have used CD69 and CD103 to identify two distinct subsets of memory T cells that are retained within these two organs in the absence of recent T cell activation. These two populations are transcriptionally distinct by S1P1 and KLF2 expression and have different anatomic distributions, with a selective retention of EBV-specific T cells in tonsillar tissues suggesting that they are strategically positioned at sites of possible antigen encounter. Our data also indicated the important roles for two locally-produced cytokines, IL-15 and TGF-β, in determining tissue residence of CD8^+^ T cells.

## Results

### CD69 is expressed on memory CD8^+^ T cells in the absence of recent T cell activation

**Fig 1:**
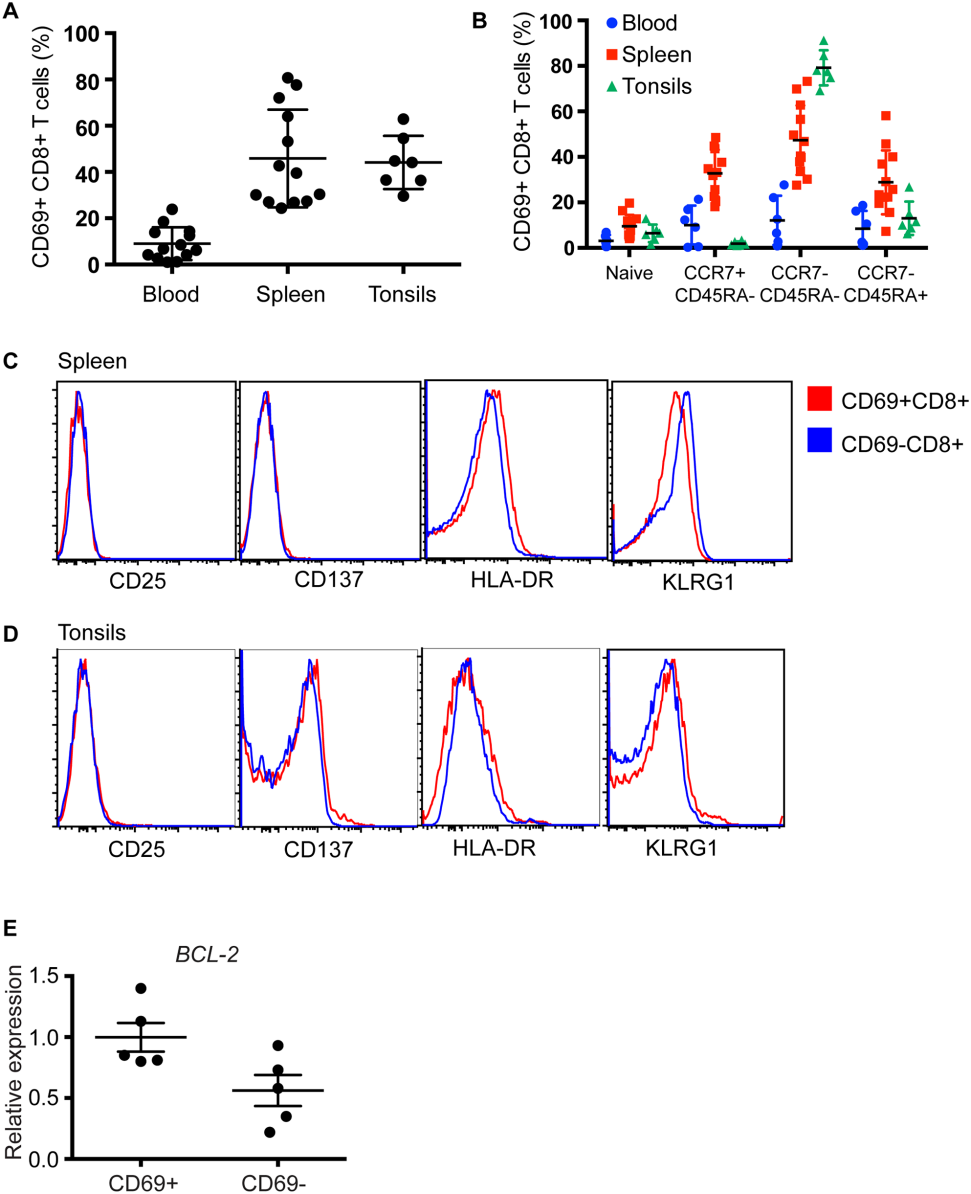
CD69 is expressed on CD8^+^ T cells in the absence of recent T cell activation. The phenotype of CD8^+^ T cells isolated from peripheral blood, spleen and tonsils were examined by flow cytometry. (A) The mean (± SD; n = 13 for blood and spleen and n = 7 for tonsils) percent of CD69^+^CD8^+^ T cells in these compartments. (B) CD69 expression on naïve (CCR7^+^CD45RA^+^), central memory (TCM, CCR7^+^CD45RA^—^), effector memory (TEM, CCR7^—^CD45RA^—^) and TEMRA (CCR7^—^CD45RA^+^) CD8^+^ T cells from blood (blue), spleen (red) and tonsils (green). Individual dots represent different donor samples. (C and D) Representative flow cytometry histogram plots show the expression levels of activation markers CD25, CD137, HLA-DR and KLRG1 between the CD69^+^CD8^+^ T cells (red) and CD69^—^CD8^+^ T cells (blue) in the spleen (C) and tonsils (D). Data is representative of 5 independent experiments. (E) Graph shows the relative expression levels of *BCL2* between CD69^+^ TEM CD8^+^ T cells and CD69^—^TEM CD8^+^ T cells from human spleen (n = 5). P = 0.0625 by one-way ANOVA.

We chose to compare circulating T cells with T cell populations isolated from human spleens and tonsils, two lymphoid tissues that are important for infections acquired through blood and the oropharynx respectively. As previously reported [28], CD69 expression was minimal on circulating T cells; however, was consistently detected on 25–75% of splenic and tonsillar CD8^+^ T cells (Fig 1A). CD69 expression was largely confined to memory CD8^+^ T cell populations, with the CCR7^—^CD45RA^—^effector memory and CCR7^—^CD45RA^+^ TEMRA subsets together accounting for over 75% of CD69^+^CD8^+^ T cells in the spleen and over 90% in the tonsils (Fig 1B). Since CD69 is also expressed on recently activated T cells, we examined CD8^+^ T cell subsets for early (CD137, CD25) or late (HLA-DR) T cell activation markers. When compared to CD69^—^CD8^+^ T cells however, there were no differences in the expression levels of these markers on CD69^+^ CD8^+^ T cells (Fig 1C and 1D). In addition, recently activated effector T cells can be identified by their high expression of KLRG1. Both CD69^+^ and CD69^—^CD8^+^ T cells in spleen (Fig 1C) and tonsils (Fig 1D) however, were largely KLRG1^low^. This demonstrated that CD69 was expressed on memory CD8^+^ T cells in the absence of recent T cell activation. Enhanced T cell survival capacity is also crucial for the persistence of memory T cells [38]. In this regard we determined the expression of the pro-survival gene *BCL-2* in purified CD69^+^ and CD69^—^effector memory CD8^+^ T cells. Compared to CD69^—^memory CD8^+^ T cells, CD69^+^ memory CD8^+^ T cells expressed higher levels of *BCL-2* (Fig 1E), suggesting that CD69^+^ memory CD8^+^ T cells are better equipped for survival than their CD69^—^counterparts. Together these data clearly demonstrate that CD69 is expressed on memory CD8^+^ T cells in these lymphoid tissues in the absence of recent T cell activation.

### Distinct subsets of memory CD8^+^ T cells populate human lymphoid tissues

**Fig 2:**
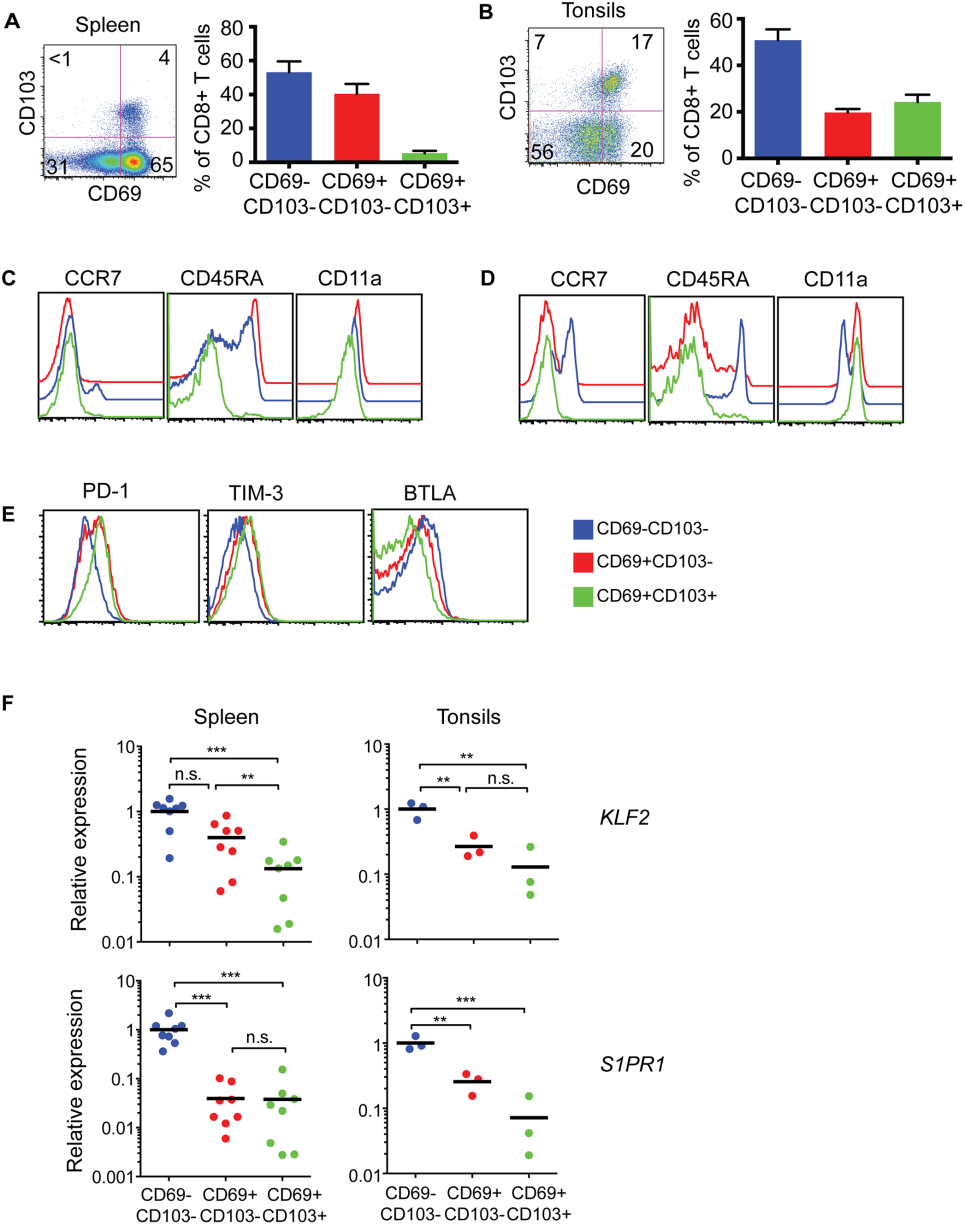
Two subsets of CD8^+^ T cells are retained within human lymphoid tissues. The co-expression of CD69 and CD103 by CD8^+^ T cells isolated from spleen and tonsils were determined by flow cytometry. (A and B) Representative flow cytometry plots (left) and graphs (right; mean ± SEM; n = 10) show the proportion of CD69^+^CD8^+^ T cells expressing CD103 in human spleen (A) and tonsils (B). (C—E) Representative flow cytometry plots showing the expression levels of CCR7, CD45RA and CD11a between CD69^—^CD103^—^(blue), CD69^+^CD103^—^(red) and CD69^+^CD103^+^ (green) CD8^+^ T cell subsets from the spleen (C) and tonsils (D) and the expression of PD-1, TIM3 and BTLA in tonsils (E). Data is representative of 3–5 independent experiments. (F) Relative expression of *KLF2* and *S1PR1* in purified CD8^+^ T subsets from the spleen (left panels) and tonsils (right panels). Individual dots represent different donor samples (n = 8 for spleen and n = 3 for tonsils). Statistical analysis was performed using one-way ANOVA and Tukey test. P<0.05 is noted with * and P<0.0005 is noted with ***.

Since at least two distinct subsets of Trm cells can be identified in mouse tissues and human skin by differential expression of CD103 [6,13,26,27,39], we also examined CD8^+^ T cells from human spleens and tonsils for co-expression of CD103 and CD69. As shown in Fig 2A and 2B, in both tissues CD103 expression was largely restricted to the CD69^+^ subset; however, while in the spleen CD103^+^ cells made up only a small fraction of CD69^+^ population, in the tonsil these cells comprised around 50% of the CD69^+^ subset. As in the mouse therefore, human lymphoid tissues contain both CD69^+^CD103^—^and CD69^+^CD103^+^ subsets of CD8^+^ T cells. To gain further insight into possible differences between these two subsets, we characterized both populations phenotypically using the T cell differentiation markers CD45RA and CCR7 (the latter known to be involved in T cell exit from peripheral tissues and T cell retention in lymph nodes [22,40]), and CD11a, the alpha chain of the integrin LFA-1 known to be associated with memory T cell retention in murine tissues [41]. As shown in Fig 2C and 2D, while CD69^+^CD103^—^CD8^+^ T cells were divergent in terms of CD45RA and CCR7 expression, the CD69^+^CD103^+^ CD8^+^ T cell subset was uniformly CD45RA^—^CCR7^—^in both the spleen and tonsils. The expression levels of CD11a varied between the organs. In the spleen there were no major differences between the three subsets, however in the tonsils both CD69^+^ subsets expressed higher levels when compared to the CD69^—^subset. Studies in mice suggested that Trm cells have high levels of PD-1 and therefore we asked whether any of the subsets in humans expressed PD-1. We also examined TIM-3 and BTLA, two markers often associated with cell exhaustion. These showed that CD69^+^CD103^+^CD8^+^ T cells had the highest level of PD-1, followed by CD69^+^CD103^-^CD8^+^ T cells (Fig 2E). Neither of these subsets however, appeared to express both TIM-3 and BTLA (Fig 2E), suggesting that they are unlikely to be exhausted cells.

To extend the analysis of phenotypic differences between CD69^+^CD103^+^ and CD69^+^CD103^—^memory cell subsets, we next assessed expression of S1P1 and KLF2, both of which are down-regulated in, and are thus a feature of Trm cells [18]. We purified CD103^+^CD69^+^, CD103^—^CD69^+^ and CD103^—^CD69^—^CCR7^—^memory CD8^+^ T cells from spleen and tonsils by sorting and then quantified mRNA levels by Q-PCR. This showed that the CD69^+^CD103^+^ subset of memory T cells had significantly down-regulated transcription of both markers, whereas the CD69^+^CD103^—^subset had down-regulated *S1PR1* substantially but *KLF2* only partially (Fig 2F). These data suggested that although both CD69^+^CD103^+^ and CD69^+^CD103^—^populations are likely to be retained within the tissues, the mechanisms that regulate their retention could be different.

### CD103^+^CD69^+^ and CD103^—^CD69^+^ CD8^+^ T cells localize to different anatomical regions within the lymphoid tissues

**Fig 3:**
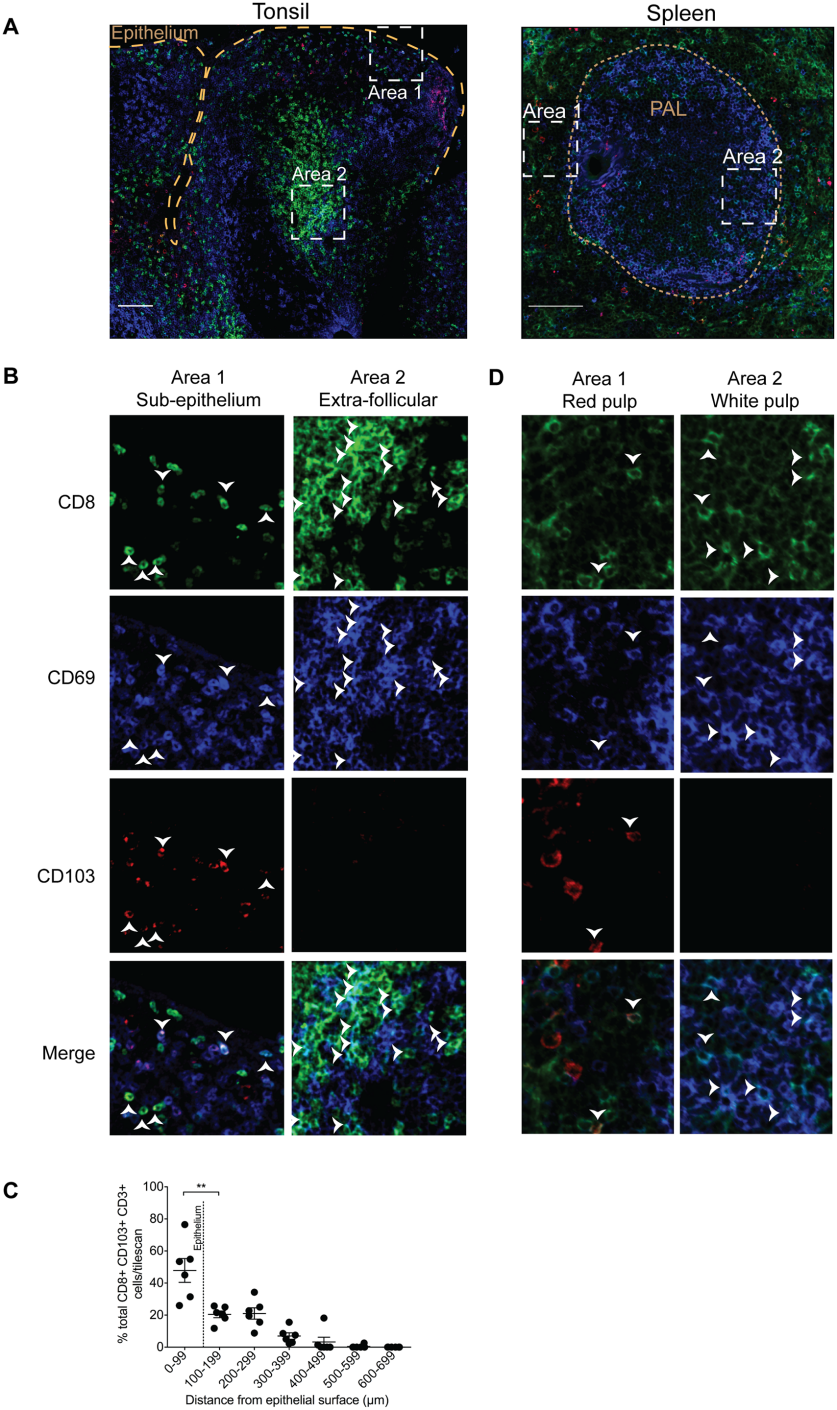
CD69^+^CD103^+^CD8^+^ T cells localize near the epithelial barrier in tonsils. The locations of CD69^+^CD8^+^ T cell subsets were determined by immunohistochemistry. (A) Immunofluorescence microscopy images of human tonsils show the localization of CD8 (green), CD69 (blue) and CD103 (red). Scale bar represents 100 μm. (B) Higher magnification of areas 1 & 2 show the localization of CD103^+^CD69^+^CD8^+^ and CD103CD69^+^CD8^+^ T cells within the tonsils. (C) White arrowheads point to CD103^+^CD69^+^CD8^+^ T cells in areas 1 and CD103^—^CD69^+^CD8^+^ T cells in area 2. (C) Quantitative analysis of the distance of CD103^+^CD3^+^CD8^+^ T cells from the epithelium shows majority localizing near the epithelial surface (P = 0.0022 by two-tailed Mann Whitney U-test). (D) Immunofluorescence microscopy of human spleen sections shows the localization of CD8 (green), CD69 (blue) and CD103 (red). Scale bar represents 100 μm. Higher magnification of areas 1 and 2 show the distribution of CD103^+^CD69^+^CD8^+^ and CD103^—^CD69^+^CD8^+^ T cells. White arrowheads in area 1 show the CD103^+^CD69^+^CD8^+^ T cells and in area 2 show the CD103^—^CD69^+^CD8^+^ T cells.

To characterize further the differences between CD103^—^CD69^+^ and CD103^+^CD69^+^ CD8^+^ T cell populations, we examined the anatomical location of these subsets. Immunofluorescence microscopy showed clear differences in the localization of these two subsets in tonsils and spleen. Fig 3A shows the overview of the stains in tonsils and spleen. Higher magnification of tonsillor sub-epithelial region (area 1) and extra-follicular region (area 2) revealed that CD103^+^CD69^+^CD8^+^ T cells preferentially localized near the epithelial barrier surface while CD103^—^CD69^+^CD8^+^ T cells were largely localized in the extra-follicular regions (Fig 3B). Quantitative analysis of the proportion of CD103^+^ T cells near the epithelium confirmed that the majority of this subset was indeed localized near the barrier surface (Fig 3C). In the spleen there were only a few CD69^+^CD103^+^CD8^+^ T cells, and these were located within the red pulp (area 1) (Fig 3D). The CD103^—^CD69^+^CD8^+^ T cells however, were localized within the periarteriolar lymphoid sheaths (PALS) (area 2) (Fig 3D). Together this reveals that CD103^—^CD69^+^ and CD103^+^CD69^+^ CD8^+^ T cells localize to different anatomical locations within human lymphoid tissues.

### IL-15 and TGF-β transcriptionally down-regulate *KLF2* and *S1PR1*

**Fig 4:**
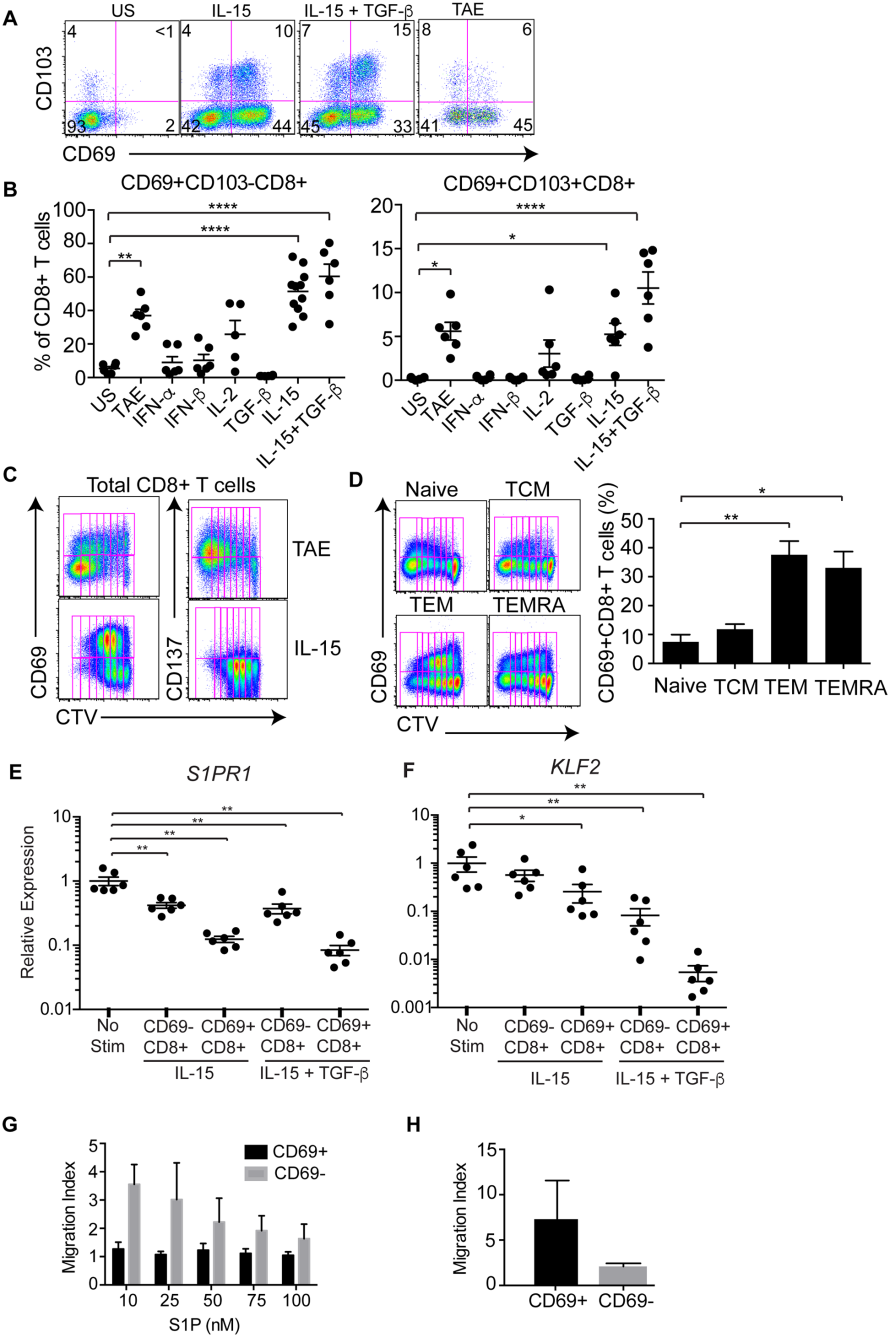
IL-15 and TGF-β co-operate to extinguish expression of *KLF2* and *S1PR1*. (A) Representative flow cytometry plots show the expression of CD69 and CD103 by CD8^+^ T cells following 7 day culture under different conditions: unstimulated (US), IL-15 or IL-15 + TGF-β and polyclonal stimulation (TAE). (B) Plot shows the proportion of CD69^+^CD103^-^ (left panel) and CD69^+^CD103^+^ (right panel) CD8^+^ T cells following 7 day culture with different cytokines. Data are represented as the mean and SEM of 5–11 donors. (C) Representative flow cytometry plots show the expression of CD69, CD137 and dilution of cell trace violet (CTV) dye following stimulation of circulating CD8^+^ T cells for 7 days with TAE beads (upper panels) or IL-15 (lower panels). (D) Representative flow cytometry plot and graph show the expression of CD69 and the dilution of cell trace violet dye following stimulation of purified circulating naïve (n = 4), TCM (n = 2), TEM (n = 8) and TEMRA (n = 5) CD8^+^ T cells for 7 days with IL-15. (E-F) Plots show the relative expression of *S1PR1* (E) and *KLF2* (F) in CD69^+^ or CD69^—^CD8^+^ T cells following culture for 7 days with no stimulation or stimulation with IL-15 with and without TGF-β. Purified circulating CD8^+^ T cells were cultured for 7 days and the resulting CD69^+^ and CD69^—^populations were purified by cell sorting. The expression levels of KLF2 and S1pr1 were quantified by RT-PCR. Individual dots represent different samples and the data is represented as the mean ± SEM. Statistical analysis was performed using one-way ANOVA and Tukey test. P<0.05 is noted with * and P<0.005 is noted with **. (G-H) The ability of IL-15 induced CD69^+^CD8^+^ T cells to migrate to S1P and CCL5 (20 nM) was tested in trans-well migration assays. Cultured cells were sorted as stated above (for F) and their ability to migrate towards different concentrations of S1P (G) or CCL5 (20 nM) (H) was determined. Data represent the mean and SEM of three independent experiments using three different donors. Statistical analysis was performed using two-way ANOVA and the p value was < 0.05.

Having identified two distinct subsets of CD8^+^ T cells that were retained within human lymphoid tissues, we wanted to determine the factors that influenced their retention. Studies in mice have shown that the maturation of Trm T cells is largely determined by cytokine exposure [1,19,27]. We therefore asked whether cytokines could potentiate the retention of CD8^+^ T cells in humans by inducing a Trm-phenotype in circulating CD8^+^ T cells. To this end, CD8^+^ T cells from peripheral blood were stimulated for 7 days either with candidate cytokines or with T cell activation and expansion beads (TAE) as a polyclonal TCR stimulus and the expression of CD69 and CD103 examined by flow cytometry. Stimulation with IL-15 alone consistently up-regulated CD69 on resting human CD8^+^ T cells (Fig 4A and 4B) including a small population of CD103^+^CD69^+^ CD8^+^ T cells (Fig 4A). By contrast, type I interferons IFN-α and IFN-β, which in mice have been shown to induce CD69 expression on T cells, and IL-2 had little effect in these experiments (Fig 4B). Although TGF-β failed to induce CD69 expression by itself, together with IL-15, TGF-β induced a small proportion of CD103^+^CD69^+^ CD8^+^ T cells (Fig 4A and 4B). Stimulation through the TCR and co-stimulatory receptors using TAE beads resulted in a large proportion of CD69^+^ and small proportion of CD103^+^ populations (Fig 4A). Importantly, in contrast to this polyclonal TCR stimulation which up-regulated activation markers such as CD137, IL-15 induced CD69 expression in the absence of CD137 expression (Fig 4C). In addition, CD8^+^ T cells isolated from the spleen not only proliferated in response to IL-15, but also maintained the expression of CD69 (S1A Fig). In order to determine which subsets of circulating CD8^+^ T cells up-regulated CD69 in response to IL-15 simulation, we purified peripheral blood naïve (CCR7^+^CD45RA^+^), central memory (TCM, CCR7^+^CD45RA^—^), effector memory (TEM, CCR7^—^CD45RA^—^), and TEMRA (CCR7^—^CD45RA^+^) CD8^+^ cells from blood by cell sorting, labeled them with cell trace violet (CTV) to track cell proliferation and stimulated them for 7 days with IL-15. This revealed that although all 4 subsets responded to IL-15 and underwent robust proliferation, expression of CD69 was mainly induced on TEM or TEMRA CD8^+^ T cells (Fig 4D). These data suggest that effector memory CD8^+^ T cells may be more responsive to IL-15-induced CD69 expression, which is consistent with the greatest proportion of CD69^+^ cells within the TEM subset in human tonsils and spleens (Fig 1B).

We next determined whether IL-15, TGF-β and IL-2 could induce the transcriptional down-regulation of *S1PR1* and *KLF2* in human CD8^+^ T cells. We stimulated peripheral blood CD8^+^ T cells with IL-15 or IL-2 in the presence or absence of TGF-β for 7 days, purified CD69^+^ and CD69^—^CD8^+^ T cells by cell sorting, isolated their RNA and determined expression levels of *KLF2* and *S1PR1* by Q-PCR. As shown in Fig 4E and 4F, IL-15 markedly down-regulated *S1PR1* transcription in the cells that had converted to CD69^+^ status, whereas its effects on *KLF2* expression were partial and amplified considerably when TGF-β was also present. Similarly, IL-2 also down-regulated both *KLF2* and *S1PR1*, however, the presence of TGF-β did not result in further down-regulation (S1B Fig). In order to determine whether IL-15 induced CD69^+^CD8^+^ T cells were unresponsive to S1P gradient we performed trans-well migration assay. This showed that the migration of CD69^+^CD8^+^ T cells towards S1P was significantly lower when compared to their CD69^—^CD8+ T cell counterparts (Fig 4G), despite being able to migrate efficiently to CCL5 (Fig 4H).

### Constitutive expression of IL-15 within lymphoid tissues can influence the retention of memory T cells

**Fig 5:**
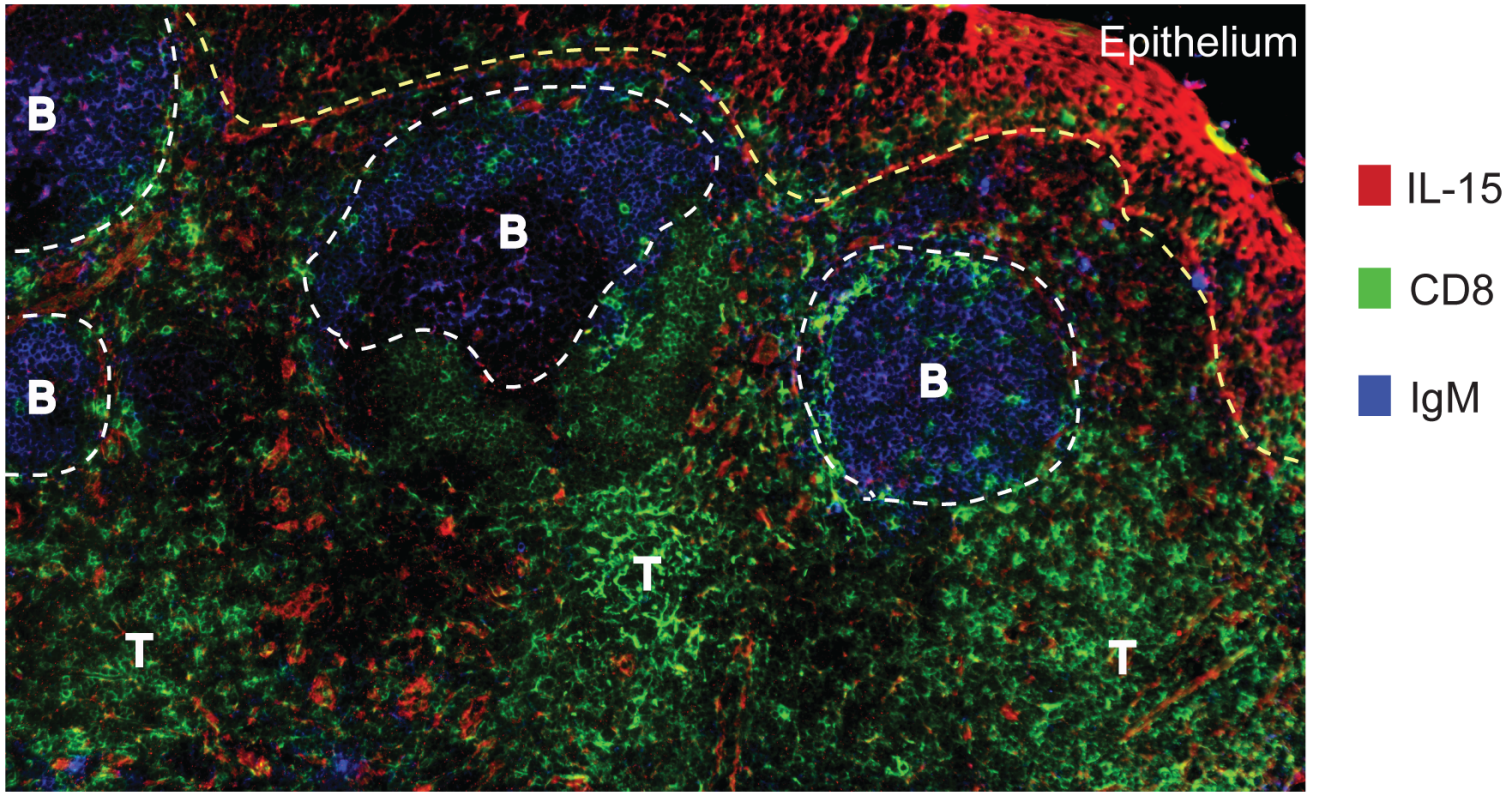
Constitutive expression of IL-15 within tonsils. Immunofluorescence microscopy of frozen section of the tonsils show the presence of IL-15 expressing cells in the T cell areas and the epithelial lining of the tissue. Sections were stained with anti-IL-15 (red), anti-CD8 (green) and anti-IgM (blue). Yellow dashed line marks the epithelial barrier surface and the white dashed-lines show B cell follicles (B). ‘T” indicates the extra-follicular regions where T cells localize.

In view of the above findings, we then examined whether IL-15 expressing cells were present within human lymphoid tissues. Frozen sections of tonsils were stained for IL-15, CD8^+^ T cells and B cells. As recently observed in IL-15 reporter mice [42], IL-15 producing cells were abundant in T cell areas of human tonsils, but largely absent from B cell follicles (Fig 5). In addition, IL-15 was detected in the squamous epithelial cells lining the tonsils. These data provide evidence for the constitutive expression of IL-15 within human tonsils.

### Selective retention of virus-specific CD8^+^ T cells within human lymphoid tissues

**Fig 6:**
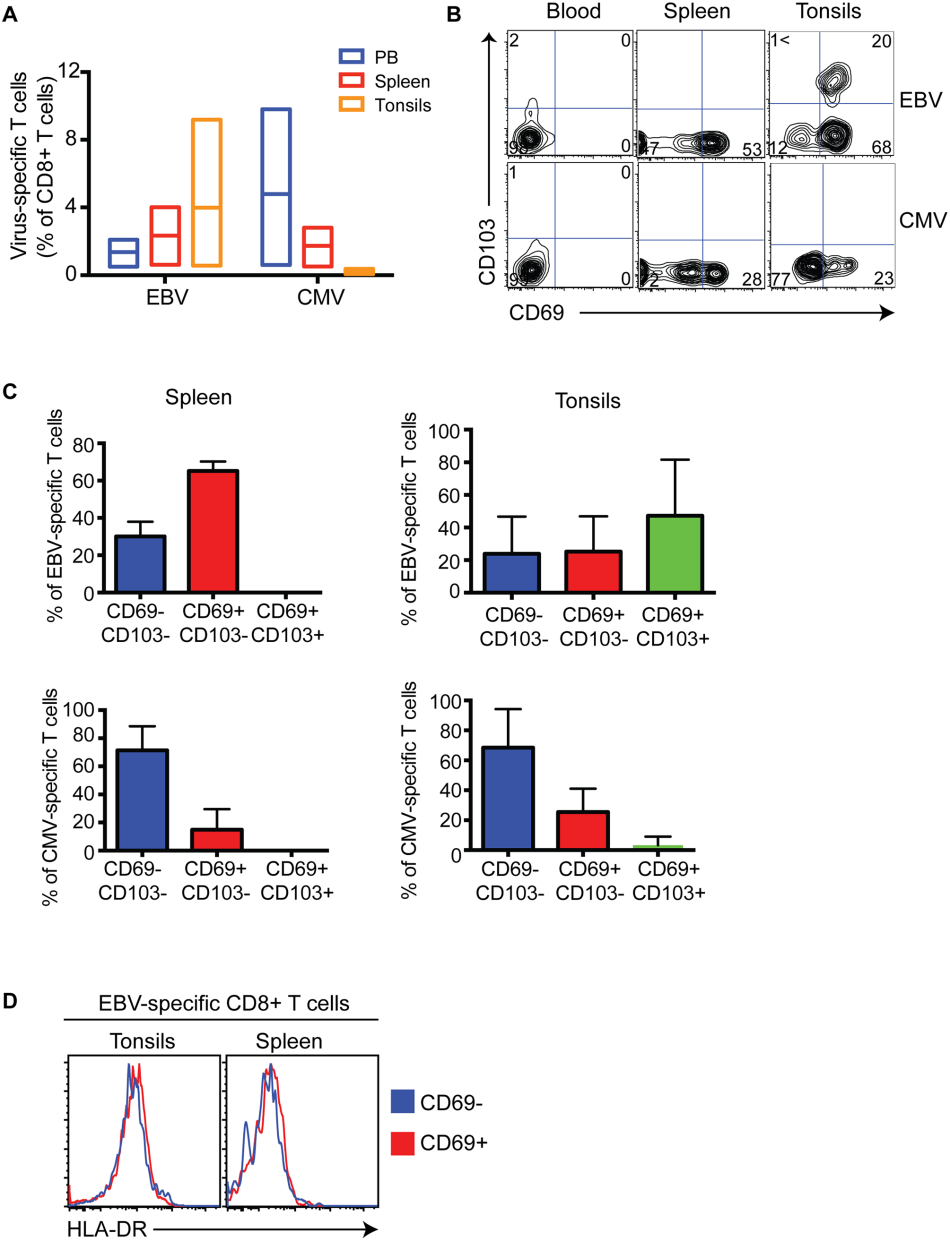
Selective retention of EBV-specific CD8^+^ T cells in tissues. Virus-specific cells were determined by staining single cell suspensions with soluble peptide-MHC complexes (dextramers). (A) The graphs show the normal range and the line at mean value for each virus-specific population in blood (blue), spleen (red) and tonsils (orange). (B) Representative flow cytometry plots show the dual expression of CD69 and CD103 on EBV and CMV-specific CD8^+^ T cells in blood, spleen and tonsils. (C) Graphs show the proportions of CD69^—^CD103^—^, CD69^+^CD103^—^and CD69^+^CD103^+^ EBV-specific and CMV-specific CD8^+^ T cells from spleen and tonsils. Data is presented as the mean ± SEM of n = 5 (for EBV specific cells in tonsils and CMV-specific cells in spleen), n = 6 (EBV-specific cells in spleen) and n = 3 (CMV-specific cells in tonsils) different samples. (D) Representative flow cytometry overlayed histogram plots show no difference in the expression of HLA-DR between CD69^—^(blue) and CD69^+^ (red) EBV-specific CD8^+^ T cells in the tonsils (left) and spleen (right).

In order to understand the significance of CD69^+^CD103^+^ and CD69^+^CD103^—^CD8^+^ T cell subsets that persist in human lymphoid tissues, we examined the tissue distribution and phenotype of CD8^+^ memory T cells specific for two common viral pathogens, the B-cell tropic EBV and largely myeloid cell-tropic CMV. The typical values, including that of some paired blood-tissue samples, of the proportion of EBV and CMV-specific CD8^+^ T cells in the blood, spleen and tonsils suggested that EBV-specific CD8^+^ T cells preferentially accumulated in the tonsils, while CMV-specific CD8^+^ T cells remained in the blood (Fig 6A). This prompted us to examine the phenotype of the virus-specific cells in these tissues to determine the proportion of Trm cells. While both EBV-specific and CMV-specific CD8^+^ T cells in circulation were CD69^—^CD103^—^(Fig 6B), a significant proportion of EBV-specific and a small proportion of CMV-specific CD8^+^ T cells in the spleen expressed CD69 (Fig 6B and 6C). Strikingly, neither EBV nor CMV-specific CD8^+^ T cells in the spleen expressed CD103 (Fig 6B and 6C). By contrast, large proportions of EBV-specific CD8^+^ T cells in the tonsils were CD69^+^CD103^+^ and the small fraction of CMV-specific cells found in the tonsils remained CD69^—^CD103^—^(Fig 6C). The expression levels of late activation marker, HLA-DR was similar between CD69^+^ and CD69^—^EBV-specific cells in the tonsils (Fig 6D), suggesting that the CD69 expression was not due to recent T cell activation. These observations revealed that there was selective accumulation of CD69^+^CD103^+^ EBV-specific CD8^+^ T cells in the tonsils.

## Discussion

Maintenance of memory CD8^+^ T cells at appropriate anatomical sites appears to be crucial for optimal protection against recurrent virus infections. In this study we demonstrate that two distinct subsets of memory CD8^+^ T cells, expressing markers of tissue residence but not of recent activation, are retained within human lymphoid tissues such as tonsils and spleen. These two subsets are not only phenotypically distinct, but also anatomically separate within the tissue environment. CD8^+^ T cell memory to different viruses are differently distributed between the two subsets and show different patterns of tissue retention, with memory to an oropharyngeally-replicating virus acquiring a phenotype that is specifically retained at sites of possible virus reactivation within the tonsil. We also show that IL-15 and TGF-β can potentiate the retention of memory T cells in tissues.

Our initial experiments showed that CD69 is expressed on large proportions of CD8^+^ T cells in human tonsils and spleen in the absence of recent T cell stimulation. Previous studies have also reported similar high proportions of CD69^+^CD8^+^ T cells in human lymphoid tissues but left open the question whether CD69 was simply a marker of recent activation rather than tissue residence. The present study, showing that such CD69^+^ cells lack a range of T cell activation markers, makes it clear that their expression of CD69 reflects tissue retention or tissue-residency. Our data also show that this population is itself heterogeneous, with CD103 as a marker that differentiates at least two distinct subsets. CD103 is an adhesion molecule that has already been associated with Trm cells in the skin, brain, gut and reproductive tract in mice [6,7,9,13,19,25,43]. Although CD103 expression is not a requirement for Trm cell formation [27,43], its presence on the membrane enables T cells to bind to epithelial surfaces where its ligand E-cadherin is expressed [24]. We have identified crucial differences between the CD103^+^CD69^+^ and CD103^-^CD69^+^ CD8^+^ T cell populations in human lymphoid tissues. Both subsets exhibited dramatic down-regulation in expression of *S1P1*. However, while there was a corresponding strong down-regulation of *KLF2* in the CD103^+^ subset, this was only partial in the CD103^-^ subset. In line with this, the CD103^+^ subset was uniformly CCR7^—^, consistent with KLF2 also regulating expression of CCR7 [20]. In addition, the CD69^+^CD103^+^ subset preferentially localized to epithelial barrier. Therefore our data from human tissues not only demonstrates the differences between CD69^+^CD103^+^ and CD69^+^CD103^—^subsets, but also indicates that the CD69^+^CD103^+^ subset is more typical of the Trm cell populations described at barrier surfaces in mouse models.

Emerging evidence from different mouse models suggest that Trm cell formation is a two-step process [1]. The first step is the infiltration of a memory precursor population from blood into the tissue; a process that may or may not depend on the presence of antigen [7,9,12,13,27,44]. In the second step, the infiltrated precursor cells mature to become Trm cells through a process that largely depends on cytokines [19,27]. During this maturation process the CD8^+^ T cells not only express CD69 and CD103, but also substantially down regulate KLF2 and S1P1 [12,19]. To this end, IL-15 and TGF-β have been implicated in the development of Trm cells in mouse models, although the mechanism by which IL-15 enables Trm cell formation is unclear [19]. Here we provide evidence for a key role for these two cytokines in humans. Firstly, we demonstrate that IL-15 alone can up-regulate CD69, probably by down-modulating S1P1 and KLF2. Our data however, also reveals that down-regulation of KLF2 was only partial in the presence of IL-15 alone and TGF-β was required for the complete down-regulation of KLF2. IL-15 is a known growth factor for memory T cells and it enables T cells to persist in the absence of continuous antigen stimulation [45,46]. Therefore IL-15 is a likely candidate to retain and maintain a pool of memory T cells. Although it has been known that epithelial cells produce TGF-β, here we also demonstrate the presence of IL-15 producing cells at the sites where T cells are retained. We therefore propose that IL-15 and TGF-β could be the key regulators of CD69^+^CD103^+^ and CD69^+^CD103^-^ subsets in human lymphoid tissues.

Further insight into the differences between the two subsets came when the specificity of CD8^+^ T cells in human spleen and tonsils was examined. Although EBV persists within the memory B cell compartment, it largely remains as a true latent infection in these cells, and the evidence for its reactivation outside of oropharynx is limited. For example, the viral load in the spleen of healthy virus carriers was 20-fold lower than that observed in their peripheral blood [47], suggesting that it is unlikely that regular viral replication takes place at sites such as the spleen. By contrast, the lymphoid tissues associated with the Waldeyer’s ring in the oropharynx are likely sites where virus reactivation takes place, probably initiated when virus-infected B cells infiltrate these tissues and the infection switches from latency into lytic cycle to seed foci of replication in permissive oropharyngeal epithelium [37,48,49]. This explains the occasional bouts of clinically silent virus shedding detectable in the throat washings of healthy virus carriers [36]. Such shedding is clearly under some form of T cell control, since levels of shedding are significantly raised in T cell-compromised individuals [36,50]. In this context the phenotype of EBV-specific CD8^+^ T cells in the tonsils suggests that more than a third of them were CD69^+^CD103^+^ and therefore are likely to be positioned near the epithelial barrier where EBV reactivates. Furthermore, the CD103^+^CD69^+^ EBV-specific CD8^+^ T cells were absent in the spleen and the few CMV-specific CD8^+^ T cells that were retained in the tonsils and spleen were largely CD103^—^.

Our data reveal that CD103^+^CD69^+^ EBV-specific CD8^+^ T cells are selectively retained at sites of possible antigen encounter, which is consistent with the Trm T cell function implicated in mouse models [10]. Accumulation of virus-specific CD8^+^ T cells at sites of viral replication has been observed in other infections as well [11,34]. Here we show the distinct compartmentalization of different virus-specific resident memory CD8^+^ T cells that could be a crucial strategy for sustained protective immunity to pathogens. Our study also provides evidence for locally produced cytokines to potentiate the formation and positioning of Trm T cells. Taking together, the evidence we provide here suggest that the development of EBV-specific resident memory CD8^+^ T cells within the tonsils is likely to be influenced by the virus and the local environment. During active EBV replication, virus-specific effector CD8^+^ T cells are recruited to the tonsils. These infiltrating EBV-specific CD8^+^ T cells do not express CD103 [51]. Overtime, under the influence of IL-15 and TGF-β these effector cells are likely to mature to become CD69^+^CD103^+^ resident memory T cells, positioned along the epithelial barrier. This is supported by the finding that CD103^+^ EBV-specific CD8^+^ T cells within the tonsils only appear during convalescence [51]. The positioning of CD69^+^CD103^+^CD8^+^ T cells is likely to be facilitated by presence of E-cadherin at these sites [52]. More importantly, when compared to their circulating counterparts these CD103^+^ EBV-specific CD8^+^ T cells were highly reactive against EBV antigens [52], suggesting that the primary role of these Trm cells could be to prevent or limit the viral replication at these sites. This strategic positioning of EBV-specific Trm cells is reminiscent of herpes simplex virus (HSV)-specific Trm cells in human skin. A recent study has demonstrated that similar persistence of HSV-specific CD8^+^ Trm cells at sites of viral reactivation is crucial for the containment of the virus [33]. Therefore the fact that EBV reactivation is often asymptomatic or sub-clinical could be largely due to this effective control at the sites where the virus reactivates.

## Materials and Methods

### Human tissues and blood samples

Buffy coats from healthy blood donors along with spleens and paired blood samples from cadaveric organ donors were obtained from the Australian Red Cross Blood Services. Tonsils were obtained from patients undergoing routine tonsillectomy. Spleen and tonsil specimens were disaggregated to single-cell suspensions and the mononuclear cells were isolated using the standard Ficoll separation method. Cells were either used fresh or cryopreserved in liquid nitrogen for later work. All human experiments were approved by jurisdictional ethics committees in Sydney as well as the institutional review boards.

### Ethics statement

Approval for this study was obtained from human ethics committees of the Royal Price Alfred Hospital, St Vincent’s hospital and Sydney South West Area Health Services (Australia). Informed written consent was obtained from next of kin.

### Flow cytometry analysis

Mononuclear cells from blood, spleen and tonsils were stained with fluorochrome-conjugated antibodies (mAbs) specific for cell surface proteins. The following mAbs were used for identification of CD8+ T cells and the determination of their phenotype; anti-CD3, anti-CD8 (Biolegend), anti-CCR7 (R&D Systems), anti-CD45RA (BD Biosciences), anti-CD69, anti-CD25, anti-CD137, anti-HLA-DR, anti-CD103, anti-KLRG-1 and anti-CD11a (all obtained from Biolegend). Fluorochrome-conjugated HLA class I dextramers (Immudex) were used to identify virus-specific CD8+ T cells. EBV-specific CD8+ T cells were identified with dextramers specific for the following viral epitopes; GLCTLVAML (derived from EBV-lytic protein BMLF1), CLGGLLTMV (derived from EBV-latent protein LMP2), RAKFKQLL (derived from EBV-lytic protein BZLF1) and FLRGRAYGL (derived from EBV-latent protein EBNA3A). CMV-specific CD8+ T cells were identified with dextramers specific for the following viral epitopes; NLVPMVATV, TPRVTGGGAM, RPHERNGFTV (all derived from pp65 protein), VLEETSVML, ELRRKMMYM, ELKRKMMYM (all derived from IE-1 protein) and VTEHDTLLY (derived from pp50 protein). Stained cells were analyzed on either FACSCanto II or LSRFortessa flow cytometer (BD Biosciences) and the data processed using FlowJo software (Treestar, Ashland, USA).

### Quantitative PCR analysis

RNA was isolated immediately after *ex vivo* purification of T cells or from cells after 7 days of culture using RNeasy kit (Qiagen). Total RNA was then reverse transcribed with oligo-dT. For *BCL2*, the following Real-time PCR primer set was used; forward, 5’–ttgacagaggatcatgctgtactt– 3’ and reverse, 5’–atctttatttcatgaggcacgtt- 3’. Q-PCR was performed with assorted commercially available Taqman assays (Hs00824723_m1, Hs00984230_m1, Hs02800695_m1, Hs00173499_m1, Hs00360439_g1) and Taqman Fast Advanced Mastermix on a StepOnePlus Real-Time PCR cycler (Life Technologies). The threshold cycle of *S1PR1* and *KLF2* for each cell population was normalized to the arithmetic mean of *HPRT*, *B2M* and *UBC* housekeeping genes (ΔCt). Normalized gene expression of each cell type was compared to the gene expression of a reference population with expression set to 1 according to the 2^(-ΔΔCT)^ method.

### Immunohistochemistry

Frozen sections of the tonsils and spleens were acetone-fixed and stained for different markers with mAbs using standard protocols [25]. The following primary mAbs were used to identify lymphocytes, anti-CD8 (Abcam), anti-CD3 (Biolegend/ AbD Serotec) (to reveal CD8+ T cells) and anti-IgM (Life technologies) (to reveal B cells). The expression of CD69 and CD103 was identified using purified, flurophore-conjugated or biotin-conjugated anti-CD69 mAb (BioLegend) and anti-CD103 mAbs (BD Biosciences). The presence of IL-15 producing cells was determined using anti-IL-15 mAb (Abcam). The following secondary antibodies were used to reveal specific staining; affinity purified F(ab’)_2_ fragments of AF647 or AF488 or Cy^TM^3 conjugated donkey anti-mouse IgG and Cy^TM^3 conjugated donkey anti-rabbit IgG (all obtained from Jackson ImmunoResearch). Control antibodies or secondary mAbs in the absence of primary mAbs were used to determine background fluorescent levels. Images were acquired using Delta Vision Personal (Olympus) or Zeiss LSM700 microscope and analysed Imaris software (Bitplane).

### Cell isolation and *in vitro* cytokine stimulation

CD8^+^ T cells were isolated from PBMCs using magnetic separation kit (Dynal). Isolated cells or total PBMCs were cultured for 7 days in the presence of cytokines (50 ng/ml of IL-15, IFN-α, IFN-β, TGF-β and 50 U/ml of IL-2) or with T cell activation and expansion beads (TAE; anti-CD3/CD28/CD2 mAb micro beards, Miltenyi Biotech; Polyclonal stimulation). Different doses of IL-15 (1, 10 or 50 ng/ml) were used for stimulation of virus-specific CD8^+^ T cells. For experiments where T cell proliferation was measured, 1–2 x 10^6^ purified CD8+ T cells were labeled with CellTrace Violet (CTV; Invitrogen) prior to cell culture. The proliferative history was determined based on the dilution of CTV of the T cells after stimulation.

### Migration assay

Sorted CD69^+^ and CD69^—^CD8^+^ T cells were washed in RPMI with 0.05% fatty-acid-free BSA (Sigma) and tested for transmigration across gelatin coated 5 μm transwell filters (Corning) for 4 hours to Shingosine-1-phosphate (S1P) (Sigma) or CCL5 (R&D Systems). Migrated cell numbers were enumerated by flow cytometry.

## Supporting Information

S1 Fig CD69 expression is maintained during IL-15-induced proliferation. (A) Flow cytometry plots show the expression of CD69 and CTV following culture of splenic CD8+ T cells with either TAE beads (polyclonal stimulation) or IL-15 for 7 days. (B) Plots show the relative expression of *S1PR1* (left panel) and *KLF2* (right panel) in CD69^+^ CD8^+^ T cells following culture for 7 days with no stimulation or stimulation with IL-2 with and without TGF-β. Purified circulating CD8^+^ T cells were cultured for 7 days and the resulting CD69^+^ populations were purified by cell sorting. The expression levels of *KLF2* and *S1PR1* were quantified by RT-PCR. Individual dots represent different samples and the data is represented as the mean ± SEM.
(TIF)
