# Supplementary figures and images for: Compartmentalization of Total and Virus-Specific Tissue-Resident Memory CD8+ T Cells in Human Lymphoid Organs
Source: PLoS Pathog. 2016 Aug 19;12(8):e1005799. doi: 10.1371/journal.ppat.1005799 (PMC4991796; doi:10.1371/journal.ppat.1005799)

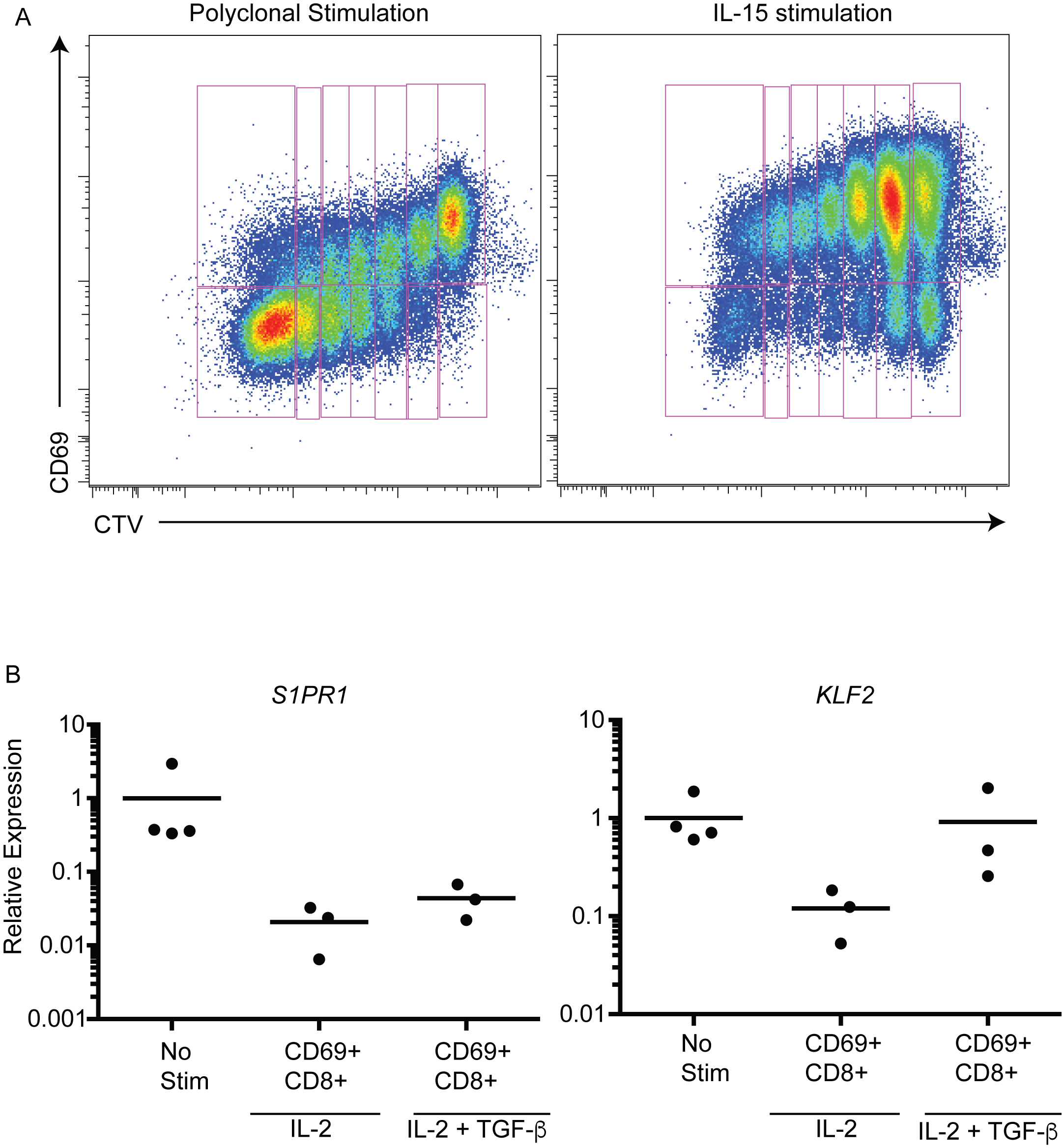

Supplement: S1 Fig — (A) Flow cytometry plots show the expression of CD69 and CTV following culture of splenic CD8+ T cells with either TAE beads (polyclonal stimulation) or IL-15 for 7 days. (B) Plots show the relative expression of S1PR1 (left panel) and KLF2 (right panel) in CD69+ CD8+ T cells following culture for 7 days with no stimulation or stimulation with IL-2 with and without TGF-β. Purified circulating CD8+ T cells were cultured for 7 days and the resulting CD69+ populations were purified by cell sorting. The expression levels of KLF2 and S1PR1 were quantified by RT-PCR. Individual dots represent different samples and the data is represented as the mean ± SEM. (TIF) [file ppat.1005799.s001.tif]
